# The Need for Consistency with Physical Laws and Logic in Choosing Between Competing Molecular Mechanisms in Biological Processes: A Case Study in Modeling ATP Synthesis

**DOI:** 10.1093/function/zqac054

**Published:** 2022-10-14

**Authors:** Sunil Nath

**Affiliations:** Department of Biochemical Engineering and Biotechnology, Indian Institute of Technology Delhi, New Delhi 110016, India

**Keywords:** ATP synthesis, molecular mechanisms, electrical neutrality, two-ion vs. single-ion theory of ATP synthase, Mitchell’s single-ion chemiosmotic theory, Nath’s two-ion theory of energy coupling and ATP synthesis

## Abstract

Traditionally, proposed molecular mechanisms of fundamental biological processes have been tested against experiment. However, owing to a plethora of reasons—difficulty in designing, carrying out, and interpreting key experiments, use of different experimental models and systems, conduct of studies under widely varying experimental conditions, fineness in distinctions between competing mechanisms, complexity of the scientific issues, and the resistance of some scientists to discoveries that are contrary to popularly held beliefs—this has not solved the problem despite decades of work in the field/s. The author would like to prescribe an alternative way: that of testing competing models/mechanisms for their adherence to scientific laws and principles, and checking for errors in logic. Such tests are fairly commonly carried out in the mathematics, physics, and engineering literature. Further, reported experimental measurements should not be smaller than minimum detectable values for the measurement technique employed and should truly reflect function of the actual system without inapplicable extrapolation. Progress in the biological fields would be greatly accelerated, and considerable scientific acrimony avoided by adopting this approach. Some examples from the fundamental field of ATP synthesis in oxidative phosphorylation (OXPHOS) have been reviewed that also serve to illustrate the approach. The approach has never let the author down in his 35-yr-long experience on biological mechanisms. This change in thinking should lead to a considerable saving of both time and resources, help channel research efforts toward solution of the right problems, and hopefully provide new vistas to a younger generation of open-minded biological scientists.

## 1. The Inviolability of Electroneutrality

**Figure 1.**
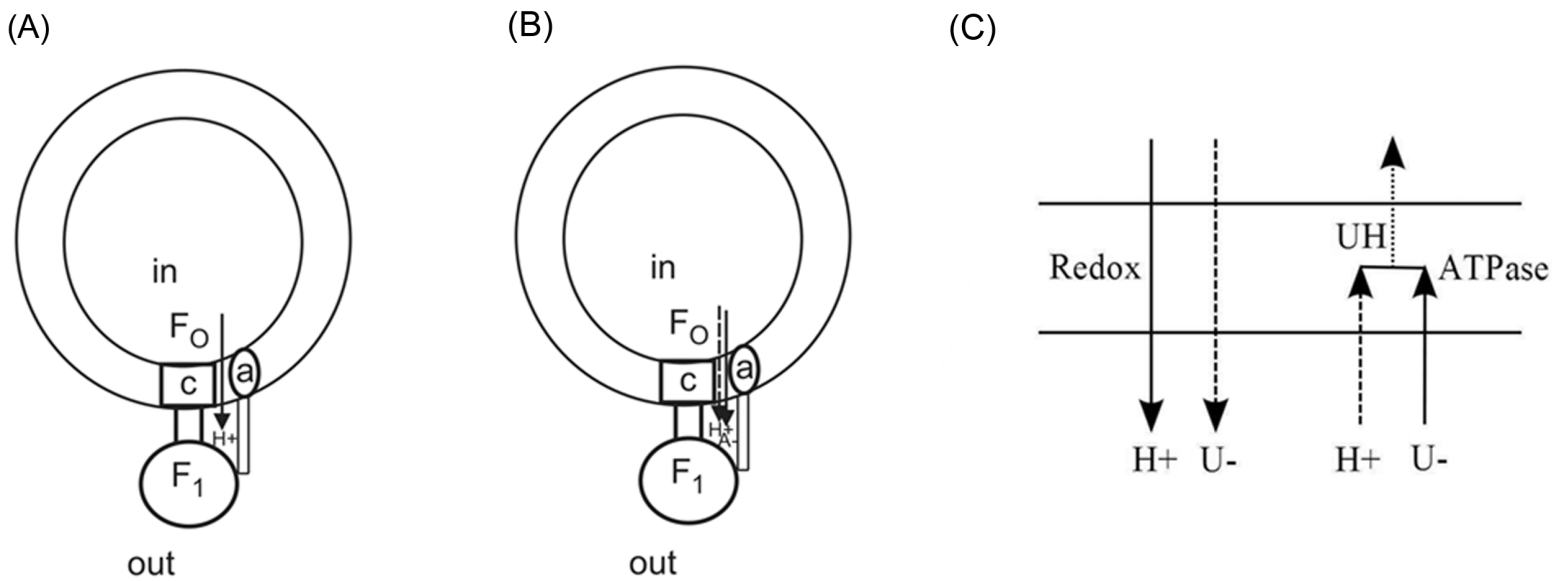
(A), (B) Representation of models of energy coupling in ATP synthesis in biochemical systems of solely purified F_0_F_1_-ATP synthase reconstituted into phospholipid membrane vesicles, for example, in a succinate bath and shown to yield physiological rates of ATP synthesis^3–7^—see also the paper by Nath and Elangovan,^62^ and the pioneering work of Jagendorf and Uribe.^66^**Scheme based on Mitchell’s chemiosmotic theory**(A), and **Nath’s two-ion theory of energy coupling and ATP synthesis**(B). The co-anion transport of succinate (A^–^) in the mitochondrial enzyme in (B) may be replaced by counter-cation transport of K^+^ or Na^+^ in a direction opposite to H^+^ translocation. The ion translocations in (B) are not simultaneous, but are ordered and sequential, and therefore lead to the generation of a *local* Δψ in the hemichannels of the ATP synthase. Bold arrows denote primary translocations while dashed arrows denote secondary translocations on the ATP side; the order of the translocations is reversed on the redox side. Scheme (A) makes ATP, generates a delocalized electrical potential (negative inside) by incessant H^+^ translocation against its own prohibitive electrostatic energy barrier (quantified in Nath, 2021)^17^ and violates the electroneutrality of bulk aqueous phases during sustained ATP synthesis, and is therefore incorrect. In scheme (B), this violation is corrected; the principle of overall electrical neutrality is maintained during ion transport and ATP synthesis by the *coupling* of proton and anion/counter-cation transport, and the energy of *both* ion translocations is harnessed to synthesize ATP.^15,16^ The generation of the local Δψ is a means for energy transduction and rotation in ATP synthase.^11,13^ (C)**Mechanism of uncoupling by the classical anionic uncouplers of OXPHOS** (eg, members of classes of phenols, phenylhydrazones, imidazoles, and dicoumarols, etc.), which are lipophilic weak acids. According to the two-ion theory, the weak acidic uncouplers of OXPHOS (such as 2,4-dinitrophenol and FCCP) uncouple by competing with the physiological dicarboxylic acid anion (succinate, HA^–^) for entry [U^–^ in (C)], formation of the uncharged species UH at an intermediate stage due to the lipid solubility of the uncoupler, and leave as uncharged acid. In contrast, succinate monoanion and proton translocate through the F_0_ portion of ATP synthase as individual ions (B) throughout the process of coupling, and do not recombine in the membrane as for uncoupling in (C). A unified kinetic model of both coupling and uncoupling based on the *same* mathematical framework and incorporation of the different conditions during the coupling/uncoupling process has been formulated and experimentally validated.^67,68^

A central, fundamental issue relates to the overall electroneutrality of bulk aqueous phases in single-molecule reconstitution experiments (Figure 1). Reconstitution procedures in oxidative phosphorylation (OXPHOS) with the F_0_F_1_-ATP synthase enzyme purified and incorporated into phospholipid membranes were first introduced in the pioneering work by Racker^1,2^ and the procedures further improved by several groups.^3,4^ Physiological ATP synthesis was demonstrated with the F_0_F_1_-ATP synthase purified and reconstituted into liposomes.^1–4^ The technology has been perfected over the years to enable studies with embedded single molecules of the ATP synthase.^5–7^ Mitchell’s single-ion chemiosmotic theory^8,9^ postulates that solely H^+^ flow occurs in such a reconstituted system (Figure 1A). This violates the overall electroneutrality of both the inside and outside bulk aqueous phases.

In a publication^9^ in 1969, Mitchell tried to prove that bulk-phase electroneutrality can be readily violated (see especially p. 178 of ref. 9). However, we have proved by direct calculations using the first principles of calculus and electromagnetism that Mitchell reached this odd conclusion by violation of Gauss’s law of electric fields.^10^

Proposed biological mechanisms (Figure 1A) cannot contravene physical laws.^10^ In our view, maintenance of overall electrical neutrality of bulk aqueous phases during ion translocation by membrane transporters is inviolable. Therefore, since inception, Nath’s two-ion theory of energy coupling and ATP synthesis includes a flow of anions in the same direction as the protons (Figure 1B) or an opposite flow of counter-cations (eg, K^+^ or Na^+^) such that overall electroneutrality of bulk aqueous phases is maintained.^11–17^ Both type of ion movements contribute to the energetics of ATP synthesis.^10,11,17,18^ Such a system has been shown to accurately quantify biochemical data on ATP synthesis by reconstituted F_0_F_1_-ATP synthase *without using adjustable parameters*.^16^

Uncompensated translocation of a single type of ion (Figure 1A) in an electrogenic process builds up charge that acts as an electrostatic/energy barrier to continued ion movement, and the process will thereafter cease to operate.^17^ A compensating charge flow obligatorily needs to be present in Nature (Figure 1B; see refs. 7 and 9 for an opposite view). Examples abound. The rechargeable lithium battery^19,20^ will only deliver power when two charged flows operate, that is, an Li^+^ ion flow and a compensating e^–^ charged flow. If one of the flows is inhibited, the other coupled flow stops too; a single flow cannot sustain operation of the electrochemical device. Similarly, it is not possible to build a semiconductor device with solely electron flow; both electrons and holes are needed.^17,21^ The prohibitive energy barriers to incessant flow of a single ion-type in membrane transporters has been quantified by Nath based on the Kirkwood–Tanford–Warshel electrostatic theories.^17^

So an important question arises: ***how is it that overall electroneutrality is being violated in the physiological reconstituted single molecule ATP synthase experiments (******Figure 1******A)?***

## 2. Absence of a Delocalized Potential in the Reconstituted ATP Synthase Experiments

The purified F_0_F_1_-ATP synthase reconstituted into phospholipid membranes (Figure 1) *does not contain any redox Complexes*. Thus, physiological ATP synthesis has been routinely shown in clean biochemical systems in which the redox agents claimed to create the *delocalized* electrical potential Δφ (Δ${\psi _m}$ in ref. 7), an essential driving force for ATP synthesis according to the classical theory,^8^ are not even present. Therefore, we concluded:^10,11^

Either no electrical potential exists (Tedeschi’s view),^22^ or the electrical potential is intra-access channel, within the F_0_-portion of the ATP synthase enzyme, that is, the Δψ is local (Nath’s view).^16^ Since H^+^ translocation due to ΔpH donates only part of the energy required to synthesize ATP, the rest has to be provided by a locally present but independent source of Δψ. Many research groups have proposed an electroneutral transport of ions^23,24^; however, we have postulated a *dynamically electrogenic but overall electroneutral ion transport* involving membrane-permeable anions (such as succinate), and protons. In our view, *mechanistically important ion binding* occurs within the profile of the electric field. Thus, through cation binding *within* the electrostatic potential field created by the translocation of anions, the enzyme is able to harness the energy of both the delocalized ΔpH and the localized Δψ.^10,11,16,17^

Hence, it is a misunderstanding to label Nath’s mechanism as “Nath’s electroneutral ‘two-ion’ theory of energy coupling” (which it is not, see above paragraph) as has often been done.^25^ Nor is the interpretation correct that the “localized Δψ (within the ATP synthase complex itself) *electrostatically attracts H^+^*, enabling translocation of H^+^ and binding to the c-ring (producing torque etc.),”^25^ as chemiosmotic ideas have been mixed up with the author’s conceptions. Why should the local Δψ, created by the anion drive H^+^ movement when H^+^ has its own electrochemical gradient, $\Delta \tilde{\mu}_{\rm {H}}$ as driving force? In this mechanism, the overall driving forces for ATP synthesis, $\Delta \tilde{\mu}_{\rm {H}}+\Delta \tilde{\mu}_{\rm {A/C}}$, or equivalently, ΔpH and ΔpA/C (or because the driving force has to change form to act, ΔpH and Δψ_A/C_, or Δψ_H_ and Δψ_A/C_, depending on the stage of the conformational cycle and where one draws the boundary surface), are created by two *independent* sources of energy, and *both* are required for rotation.

The internal matrix K^+^ concentration of isolated mitochondria (∼150 m m) closely approximates that in the cytosol; hence, postulating K^+^ as the physiological second ion across inner mitochondrial membranes open to the cytosol is problematic.^7^ Instead, a major translocative function of mitochondria ought to be the maintenance of a transport flux of *anionic* metabolites. Since Nath’s mechanism works with either a counter-cation *or* a co-anion (e.g., succinate) translocated by OXPHOS Complexes located across the ***cristae*** membranes, there is no need to “develop significant, positively adaptive changes in osmotic drive” and therefore osmotic arguments made against Nath’s mechanism^25^ are inapplicable.

Other arguments made against Nath’s mechanism also lack force. Thus, using the numbering (1)–(4),^25^ a concise response to the four issues is as follows: (1) ATP synthase would behave electrogenically and show “voltage-dependent ion channel currents” in the experiments (Section 5) or even in transporter molecules if the ordered, sequential coupled ion translocations are dynamically separable. (2) The argument is valid only if the identity of all permeant ions is known beforehand, including the *unanticipated* transport of FCCP anions with K^+^ that would explain the FCCP requirement as well as the quenching of oxonol VI fluorescence by FCCP in the experiments (Section 4). Argument (3) is true only if OXPHOS uncouplers such as FCCP are assumed to be just proton conductors (see Section 4). Statement (4) is the expected observation if the correct second ion is absent in the experiments. Thus, the four lines of evidence^25^ do not constitute evidence against Nath’s mechanism if the appropriate second ion is considered along with the considerations in Sections 4 and 5, and Figure 1C. The process of cation–anion *coupling* at the membrane–water interface in the access channels has been analyzed quantitatively in microscopic detail.^17,26^

## 3. Local Δψ Versus Delocalized Δφ

A delocalized Δφ across the energy-transducing membrane in bulk aqueous phases arising from a purely electrogenic process by translocation of a single type of ion (eg, cations)—which should have been proved—has been presumed to exist.^16^ However, the methods of measurement, including in the chemiosmotic rationales^27–29^ are indirect, and have always required a second ion (eg, K^+^ in the presence of valinomycin moving opposite to the H^+^, or a membrane-permeant anion such as succinate moving in the same direction as the H^+^) in the experimental design. ***Hence, the possibility that the second ion is also translocated and contributes to energy coupling cannot be logically ruled out from the experiments*.**^18^ Thus, the experiments do not allow a distinction between Mitchell and Nath. Furthermore, the calculation of a potential ($=RTIn \,\,(K_{in}^+ / K_{out}^+)$ by use of a Nernst-type equation does not mean that a delocalized electrical potential Δφ existed *before incubation with valinomycin*, as inferred by the rationales. In the actual system, the (local) potential created by the primary ion translocation will be immediately *collapsed* by the movement of the second ion. In fact, the presence of valinomycin should prevent the generation of a delocalized Δφ. Thus, the experiments do not provide unequivocal evidence for a *preexisting* bulk phase Δφ or protonmotive force.^18^

Above all, none of the above experiments have the power to distinguish between local and delocalized potentials. Local events in the membrane have been shown to be rapidly communicated to the bulk.^10,30^ Thus, a local potential confined to membrane access channels can always be converted into a delocalized potential across bulk aqueous phases by the subtle delusion of the indirect method of measurement. It would also involve the unanticipated destruction of all local space charge regions in the membrane^17^; hence, local mechanisms of coupling such as Nath’s are unsuspectedly eliminated from consideration even.

## 4. Other Logical Inconsistencies

**Figure 2.**
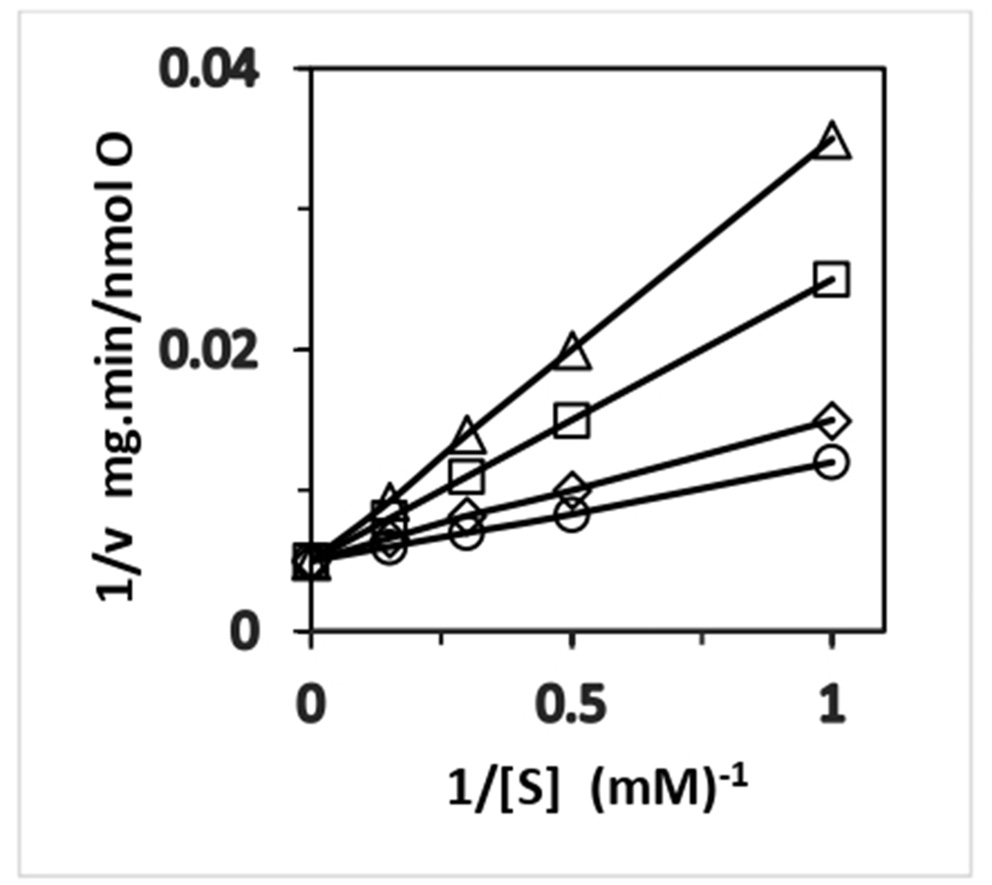
Competitive inhibition of succinate by the classical anionic OXPHOS uncoupler FCCP in mitochondria. Succinate concentration was varied between 1.0 and 6.7 m m, while the concentration of FCCP was varied in the range from 0 to 10 µm. FCCP concentrations: 0 (○); 0.5 (◊); 5.0 (□); and 10.0 µm (∆).^48^

Now, I turn from the succinate bath experiments with reconstituted ATP synthase experiments in the *absence* of anionic uncouplers in Sections 1 and 2 (Figure 1) to recent experiments in the *presence* of the anionic uncoupler, FCCP.^7^ K^+^-driven ATP synthesis in proteoliposomes in the presence of FCCP has been demonstrated under conditions when protons cannot translocate and perform useful work.^7^ However, this presents a logical inconsistency, because in the proteoliposome experiments shown in their Figures 1 and 2, the workers assume the occurrence of H^+^-translocation (inside to outside in their Figure 1, and outside to inside in Figure 2) under the very conditions where a lack of H^+^ transport has been postulated, since there is no electrochemical gradient for H^+^ movement.^7^

Hence, refuge cannot be taken in postulated H^+^ movement, under the conditions of zero H^+^ movement, which is like having the cake and eating it too. This leads to an important unanswered question: ***Upon formation of a K^+^gradient, what ion moves to maintain charge neutrality in the experiments with FCCP?***^7^

The explanation of the action of FCCP as a passive proton conductor does not collapse the delocalized Δφ/local Δψ in mitochondria; it only collapses the ΔpH. Further, it is not the only possible mechanism of uncoupling action; other uncoupling mechanisms are also possible (Figure 1C). The rates of ATP synthesis in the experiments were significantly lower in the absence of addition of the K^+^ channel openers diazoxide or pinacidil,^7^ raising questions on the role of K^+^ in a physiological context in mitochondria. Interpretation based on Nath’s mechanism removes the logical inconsistencies and helps solve the conundrum.

## 5. Channels Versus Transporters: How Have Single Transporter Currents Orders of Magnitude Below the Limit of Detectability Been Recorded?

On the order of 10^7^ ions per second permeate an open ion channel and cross the membrane. This rate is high enough for sensitive amplifiers to record the electrical current (∼1 pA) through a single channel, as first shown in pioneer work by Neher and Sakmann.^31^ However, the ATP synthase is not a channel but a membrane transporter though which the speed of ion translocation (∼10^2^–10^3^ per s) is several orders of magnitude *slower* than through open channels.^17^ Hence, even if net charge is moved across the membrane, *the electrical current generated is far too small to be detectable for a single transporter molecule*. An important unexplained technical issue is how the currents measured electrophysiologically during conduction separately of K^+^ (at $0$ mV) and H^+^ (at $+ 28$ mV) were not too small for single-molecule recording, and were therefore above the limit of detection.^7^ What factors limit the rate of ion translocation through transporter molecules in general and through the mitochondrial F_0_F_1_-ATP synthase in particular? A large number of independent biophysical studies have revealed a very small conductance for F_0_ and coupled F_0_F_1_ in the range of 0.1–1 fS.^32–36^ These studies also show that the ion currents through F_0_F_1_-ATP synthase (10^3^ s^–1^ × 1.6 × 10^–19^ C ≅ 0.1 fA) are ***fourorders of magnitude too small for single-channel recordings***. However, currents measuring between 0.5–2 pA have been reported.^7^ It should be clearly understood that if both the gates of a transporter molecule are *open simultaneously even for an instant*, then a channel-like dissipative ion flow will be recorded in electrophysiological experiments that does not reflect true transporter function.

## 6. Need to Consider the Complete Mechanism

Several deficiencies were pointed out in the theory of chemiosmosis by the founding fathers of the field.^37–39^ Nath’s work has tried to overcome these deficiencies and has studied the *complete detailed* mechanism of ATP synthesis/hydrolysis in F_0_ and F_1_, the coupling between F_0_ and F_1_ by an original torsional mechanism, including regulation and integration with ATP-utilization processes, for example, in muscle contraction.^14^,^40–43^ More than 500 journal pages have been published on these aspects. Recent work on a pure mathematical proof based on the theorems of Dirac, Ore, and Bondy–Chvátal presents a further innovation—the graph-theoretical approach shows that graphs representing energy coupling according to the chemiosmotic theory are non-Hamiltonian; therefore, chemiosmosis is an incomplete theory.^44^ The novel application of fundamental action principles of physics based on Feynman–Landau–Wheeler–Taylor paradigms^45^ to bioenergetics provides striking new insights.^46,47^ Extensive pharmacological evidence revealing site-selectivity in OXPHOS that is hardly consistent with the chemiosmotic theory has also been published recently.^48^ Hence, any analysis of Nath needs to consider the complete mechanism. By contrast, many works offer no insights on the mechanism in F_1_ or on the rotation and mechanical dynamics in the F_0_ portion of the ATP synthase, which is after all quintessentially a mechanoenzyme.

## 7. Differences between Competing Mechanisms

A mechanism of ATP synthesis proposed recently^7^ is essentially a single-ion model—only *one****type****of ion* does the work of rotation and ATP synthesis. That ion can be H^+^, K^+^, or Na^+^; any of these univalent positively charged ions will do in any proportion; they are substitutes. In particular, it was shown that H^+^ is *replaceable* by K^+^;^7^ either ion can take the work load of ATP synthesis. Hence, in that sense, it is ***not*** a *two-ion theory*, although it is called so.

On the other hand, Nath’s theory is a ***true****two-ion theory/mechanism* in that two ions are needed. Thus, for example, an H^+^ and a co-anion (like succinate)/counter-cation (like K^+^), *both* are required (separately); both their driving forces contribute energy for rotation and ATP synthesis, and their flows are *coupled* to each other. One type of ion *cannot replace* the other ion type in Nath.

Recent electrophysiological experiments^7^ reveal a variation on the chemiosmotic theme (however, for experimental *artefacts*, see Section 5). The replaceability of H^+^ by Na^+^ in several organisms had already been shown by the pioneer work of Dimroth^49,50^ and formally characterized.^51^ The model^7^ suffers from the same defects as Mitchell’s. Interestingly, Na^+^ has also been included recently as a possible substitute,^25^ in addition to H^+^ and K^+^. So should we call it a *three-ion* model? In our view, the model is only a minor extension of Mitchell’s single-ion chemiosmotic model, and we have the same objections to it as for chemiosmosis.^12,15,28^

The above discussion has summarized experimental work both old and new that has revealed the presence of cation substitutes in ATP synthesis, that is, K^+^ and Na^+^ can substitute H^+^ for catalysis of ATP synthesis in certain organisms. As per the two-ion theory, H^+^ and succinate are the physiological ions involved in coupling, while FCCP anions uncouple ion transport from ATP synthesis in the presence of H^+^ (Section 4; Figure 1C). However, the so-called “uncoupling” anion U^–^, for example, the FCCP anion, can act as a “coupling” ion with K^+^ due to its inability to form the neutral, undissociated UH form (Figure 1C) in the presence of the K^+^ cation substitute, and therefore FCCP anion and K^+^ can participate in energy coupling and enable ATP synthesis by ATP synthase.^7,48^ In other words, just as cation substitutes are possible, one can have *anion substitutes* too. Recently, we have provided biochemical evidence for the kinetically pure competitive inhibition between succinate and FCCP anions in mitochondrial OXPHOS.^48^ Hence, if succinate can permeate mitochondria or access channels in the F_0_ portion of ATP synthase, so can FCCP anions (Figure 2).

## 8. a-Subunit Access Channels

It is known that:

- Aqueous half-channels are located in the a-subunit at the a–c channel interface in F_0_.
- Protons bind to their binding site located on the c-subunit.
- Protons are involved in ATP synthesis.
  However, it has not been shown that *protons* pass through the a-subunit aqueous pathways; that is only an inference upon combining (i) and (iii) on the assumption that *only* H^+^ ions participate in energy coupling in ATP synthesis. Hence, the solution of this important issue is linked to the answer to the query posed at the end of Section 1 on the inviolability of electrical neutrality. If succinate is the second ion in mitochondrial OXPHOS, as proposed by Nath’s two-ion theory of energy coupling, then succinate can readily play the multiple roles of oxidation substrate, permeant ion, and, by binding to its site on the a-subunit access channel, as a direct activator of H^+^ translocation, due to the close physical association of the a-subunit access channel with the c-subunit access channel meant for protons, and *also* concomitantly satisfy the constraint of electroneutrality that must be *strictly* obeyed during sustained ion translocation and ATP synthesis. Hence, on this model, the detected a-subunit aqueous pathways would transport succinate anions, not protons.
  Note that even if protons did permeate the a-subunit, they would still have to also diffuse in a *transverse* direction *across* the chasm of the a–c interface in order to bind to their binding site on the c-subunit, which is unlikely. Furthermore, the a-subunit pathways are known to allow reagents >100 Da to permeate, hardly what one would expect of proton channels. The strictly conserved RLN motifs in the a-subunits of the ATP synthases of plant chloroplasts, animal mitochondria, and bacteria also point to a different function. Above all,
- Photoaffinity labeling experiments have shown that the a-subunit is selectively targeted by the triorganotin compounds.^50^
- Triorganotin compounds are known to be potent *anion* channel blockers.^52^

Logically putting together observations (iv) and (v) together, the a-subunit half-channels in F_0_ should be anion transporters, contrary to current dogma.

**Figure 3.**
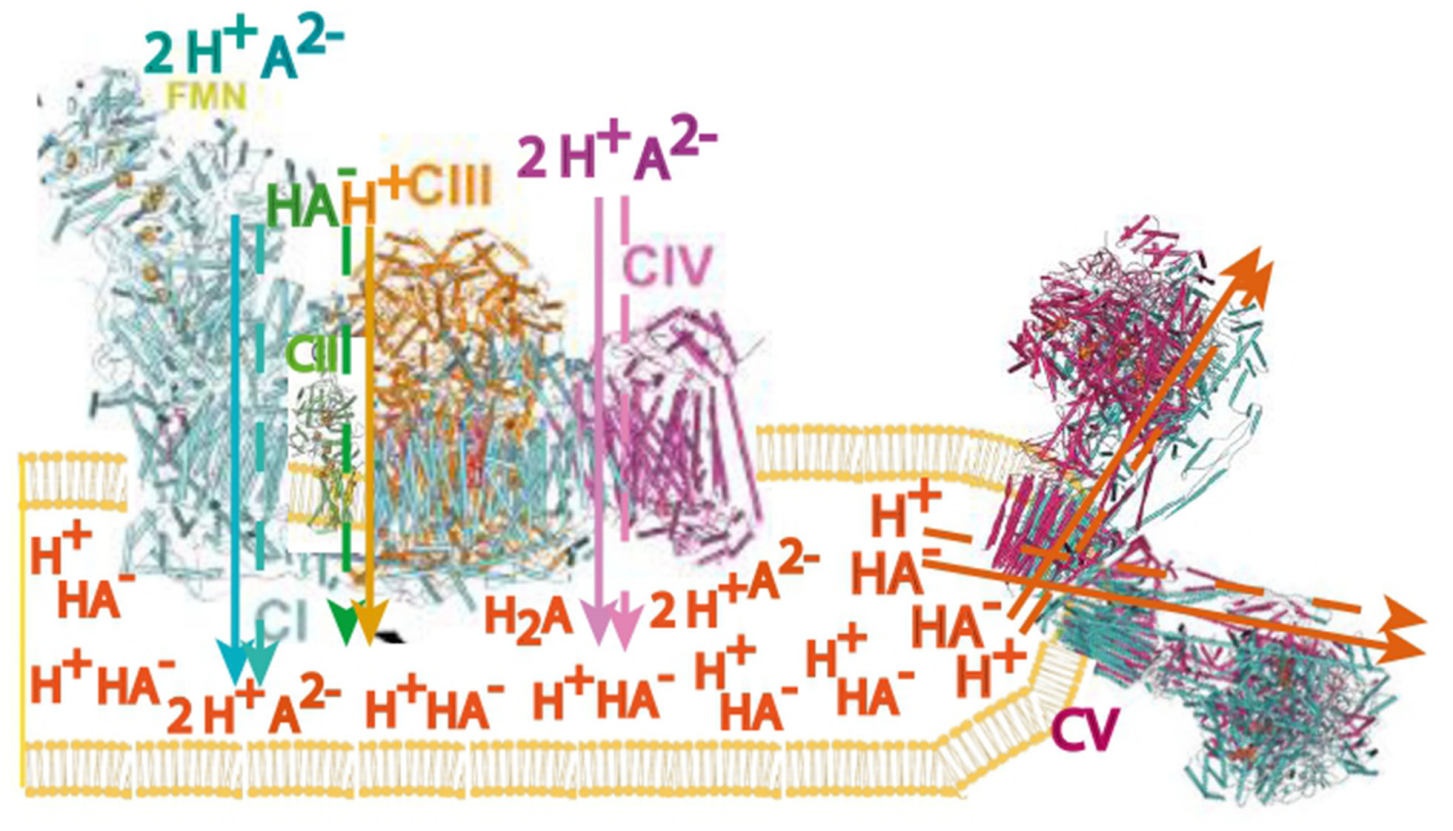
Model of supramolecular association and function of the OXPHOS Complexes I–V in the *cristae* membranes of mammalian mitochondria. The model of organization of the enzyme Complexes is integrated in the diagram with function at an overall *physiological level* based on the molecular mechanism proposed in Nath’s two-ion theory of energy coupling and ATP synthesis involving succinate anions and protons. Included in the model are advances in visualization of the internal structure of mitochondria by high-resolution scanning electron microscopy and 3D electron microscope tomography techniques,^69,70^ biochemical immunolabeling, and transmission electron microscopy.^71,72^ The structures of Complexes I–V have been assembled from the high-resolution structure of the respirasome supercomplex I_1_III_2_IV_1_ (PDB ID: 5XTH; CI in blue, CIII in gold, and CIV in magenta), the structure of Complex II (PDB ID: 1ZOY; CII in green), and the structure of the tetramer of ATP synthase, Complex V (PDB ID: 6J5K).^73^ Primary ion translocations by the Complexes I–V are shown by bold arrows, and secondary ion translocations are shown by dashed arrows. The translocation of succinate dianions and protons by Complex I and Complex IV, and of succinate monoanions and protons by Complex II–III are also depicted. The model proposes a novel *molecular* interpretation of leaks and slips. Thus [HA^–^] is the motive monoanionic form of the dicarboxylic acid that is permeant through the access channels of the ATP synthase, [H_2_A] is the neutral form that diffuses freely across the membrane ("leak"), while [A^2–^] is the dianionic form that constitutes "slip". The back-and-forth circulation of these ions from intracristal space to mitochondrial matrix is required in the new conceptual framework at the physiological level for efficient homeostasis and regulation of OXPHOS during steady state function of the system.

In summary, the F_0_F_1_-ATP synthase is a ***cotransporter***^16,17^ that catalyzes the ordered, sequential, and dynamically electrogenic but overall electroneutral transport of protons and succinate (or other anions/countercations) under physiological conditions. An overall molecular model for the functioning of Complexes I–V in mitochondrial OXPHOS according to Nath’s two-ion theory of energy coupling and ATP synthesis is depicted in Figure 3.

The OXPHOS model (Figure 3) provides a *molecular* explanation for the existence of mitochondrial leaks and slips, now identified with the neutral and dianionic forms respectively of a dicarboxylic acid anion.^53^ The model was therefore extended to include the homeostasis of the three forms—with the monoanionic form being the motive permeant form through the ATP synthase—and the regulation of the OXPHOS system based on energy demand for ATP elsewhere in the cell.^54^ This also solved the longstanding problem of respiratory control in OXPHOS,^26,54^ a problem that had not been addressed by chemiosmosis, as Chance had first pointed out and therefore requiring of a fresh solution.^39^ (The underlying reasons for the inadequacy of the chemiosmotic theory to deal with the problems of respiratory control and regulation in OXPHOS have only recently been clarified by a mathematical graph-theoretical analysis).^44^ A detailed energy audit of ATP consumption in the brain has been performed based on the above new concepts.^55^ The importance of a role for anions in ATP synthesis has been discussed by other workers also^29,56,57^; however, the identity of the coanion in oxidative phosphorylation and photophosphorylation as a dicarboxylic acid anion was due to a decade-long experimental search.^58^ Another unique aspect of the model (Figure 3) which ought to be stressed, was found later—*succinate is the only anion among the universal set of anions in nature that can deliver a monoanion: dianion ratio of 8:2* at any particular set of environmental conditions of pH, ionic strength, etc.^59^ This offers a new explanation^53–55^,^58,59^ for the consensus H^+^/2e^–^ stoichiometry of 4 for Complex I and Complex IV (ie, a total of 8 ions per 2e^–^), and H^+^/2e^–^ = 2 for Complex III (ie, 2 ions per 2e^–^) of the mitochondrial respiratory chain.^60^

## 9. New Experiments

The two-ion theory suggests several new experiments. One approach could be structural; a recent structural characterization of succinate cotransporters offers new insights.^61^ A biochemical approach that monitored the large anionic movements and traced the path of dicarboxylic acid anions in ATP synthesis during photosynthetic phosphorylation proved valuable in our hands; in this system, we could readily separate the light stage biochemistry from the dark stage biochemistry.^62^ Several variants of this general approach offer great promise on other OXPHOS systems in mitochondria and bacteria.^63,64^ An interesting third line of enquiry could characterize the *metabolic contents* of cristae and thylakoid membranes/vesicles—a beginning has already been made by Japanese groups.^65^ A gas chromatography coupled to mass spectrometry analysis of the metabolites should readily be able to detect the accumulation of dicarboxylic acids in these vesicular systems.

The key aspects discussed in Sections 1–9 can take us a long way in setting the record straighter.

## 10. Conclusions

The molecular mechanism by which biological molecular machines function has inspired an enormous amount of work in a century of research. Despite these vast efforts, it has often not been possible by experiment to arrive at the definitive molecular mechanism or to select between competing molecular mechanisms of operation of the biological motor or process. Based on a case study in modeling ATP synthesis, a new way for choosing between proposed molecular mechanisms has been advocated in this article: that of testing for consistency of the mechanisms with the known scientific laws. The imperative need to satisfy overall electrical neutrality of bulk aqueous phases during ATP synthesis in biochemically clean reconstituted systems solely containing ATP synthase—probably the most ubiquitous experimental system in the field for over 50 yr^1–7^—has been discussed (Section 1). Attempts to rationalize the opposite view of the Fiction of Electrical Neutrality compounded the error by an analysis^9^ that has since been proved to violate Gauss’s law of electric fields.^10^ Checks for logical errors and inconsistencies in proposed models has been concluded to offer another useful criterion, one that has been extensively analyzed and discussed in relation to ATP mechanism (Sections 3 and 4). It has been shown how a basic application of Ohm’s law helps identify experimental artefacts and avoid errors in interpretation (Section 5). The need to evaluate the sum total of the experimental evidence, assess all the proposed mechanisms, and consider the complete mechanism of the bioprocess have been identified as other key aspects (Sections 6–8). These criteria can help resolve the problems of choosing between proposed molecular mechanisms in bioenergetics, motility, and in other biological fields that have caused great headaches to scientists in the past. Not only would longstanding difficulties of the types detailed by historians and philosophers of science^74^ be avoided, but the progress of research in interdisciplinary scientific fields would be greatly accelerated.

## Data Availability

No new data were generated in this Evidence Review article.

## Notes

The article is organized in the following way: I analyze the complex, longstanding fundamental mechanistic issues in the field of ATP synthesis concisely (Sections 1–3), attempt to resolve the basic misunderstandings and areas of conflict (Sections 4 and 5), make differences between competing models both clear and accurate (Sections 6 and 7), and propose future experiments that can shed additional light on the subject (Sections 8 and 9).
